# Vomiting of Pregnancy—Its Etiology and Treatment

**Published:** 1891-09

**Authors:** F. Blume

**Affiliations:** Allegheny, Pa.


### VOMITING OF PREGNANCY—ITS ETIOLOGY AND TREATMENT.*

BY F. BLUME, M. D., ALLEGHENY, PA.

Pregnancy, as a rule, is complicated with a variety of disorders, which, though in many instances causing much discomfort, are termed physiological as long as they are not associated with serious disturbances of the organism. Derangements of the gastro-intestinal canal, nausea and vomiting, to the consideration of which I invite your attention to-night, are such a regular occurrence during the early period of pregnancy that experienced women consider them as positive signs of conception.

The so-called morning sickness—nausea and vomiting early in the morning, or even after meals during the first few months of gestation—have, in the large majority of cases, no effect either upon the course of pregnancy or upon the health of women. Although the ordinary morning sickness sometimes persists during the whole period of pregnancy, it remains endurable, causing the patient rather annoyance than injury. There are intermissions, either spontaneously or the consequence of some treatment, the digestive functions remain more or less normal, and the vitality of the patient is not essentially impaired. In some, fortunately very rare instances, however, nausea and vomiting become incessant and uncontrollable, the stomach rejects everything, the patient grows weaker until the most extreme degree of exhaustion is reached, and death from starvation threatens.

The onset of this grave form of the affection is gradual, and does not differ in character from the usual morning-sickness. But soon the nausea becomes more intense, the vomiting more frequent. The ejected matter consists of food, mucus and bile. The appetite is more or less impaired or perverted; the thirst is excessive; constipation is more frequent than diarrhœa; the urine is scanty, concentrated and contains albumen and casts. The

* Read before the Allegheny County Medical Society, June 16, 1891.

pulse grows small and rapid, the temperature rises and continued fever develops.

With the progress of the disease the condition of the patient becomes more and more alarming. The nausea is almost constant, adding greatly to the discomfort of the woman. The efforts at vomiting are accompanied by violent retching and pain, not the smallest amount of food or drink is retained by the rebellious stomach, the smell, even the thought of nourishment, or the slightest movement of the patient, induces an attack. The vomited matter is finally mixed with blood. The thirst is tormenting, the throat and mouth are dry, the tongue brownish, the breath fetid, the abdomen tympanitic. The consequences of this continued suffering soon become very pronounced by the marked alteration of the features, the extreme emaciation, and the profound depression of the patient. Shortly before life ends vomiting ceases and coma supervenes.

Cases of persistent vomiting, which terminate fatally, are certainly very rare. Even after the application of various methods of treatment has failed to influence the course of the disease, and while the induction of abortion was earnestly considered, the patients have recovered spontaneously, and have gone to full term, as I have seen in the only instance of this grave disorder which has come under my observation.

There is considerable diversity of opinion as to the causes which may incite hyperemesis, and, in spite of numerous theories and hypotheses, the etiology is by no means clear. It is almost universally accepted to be a reflex neurosis originating in the uterus, and dependent either upon pregnancy alone or upon coexistent pathological conditions.

Pregnancy itself, the growing ovum, which acts as an irritant by the simple mechanical distention of the uterine cavity and its peritoneal covering, is in the first place to be mentioned as the most potent etiological factor.

Cases of multiple pregnancy and hydramnion, which present a disproportion between the passive distention and the active growth of the uterus, and which frequently are complicated with hyperemesis, confirm this view. Moreover, the induction of artificial abortion, our last resource in desperate cases, which almost immediately relieves the patient when done in time, is founded upon this theory of passive uterine distention, and strongly supports it.

Spontaneous death of the fœtus, followed by immediate or remote abortion, is another remarkable fact in favor of this view.

A patient of mine, the mother of two children, was suffering from double laceration of the cervix, erosion and endometritis.

She refused surgical treatment, and was relieved by repeated irrigations of the uterus with carbolized water, and by the application of tincture of iodine. She soon afterward conceived, and her pregnancy was complicated with the ordinary morning-sickness from the second month to the beginning of the sixth, when the vomiting suddenly ceased. Two weeks thereafter she told me that she did no longer feel the movements of the fœtus, that vomiting had ceased, and that she therefore believed the child was dead. Though I could not detect the fœtal heart-sounds, I gave my opinion with reserve. Three and a half months later I delivered her of a dead fœtus about five months old.

This case affords the most striking evidence of the discontinuance of reflex symptoms after the removal of the inciting cause. We have here pregnancy complicated with pathological conditions of the uterus, as double laceration of the cervix, ectropium and probably a but partially cured endometritis, conditions which existed prior to conception and continued after the death of the fœtus. But in spite of the persistence of these pathological conditions of the uterus, and of the retention of the dead fœtus for almost four months, the vomiting disappeared with the death of the fœtus, that is, with the cessation of the mechanical distention of the uterine cavity.

The influence of primary gravidity is demonstrated by the fact that hyperemesis in its grave forms is essentially an affection of primiparous women, and it is to be referred to the greater resistance of the virginal uterus.

Numerous other causes are given as etiological factors by different observers, among them: pathological conditions of the cervix, chronic metritis and endometritis, displacements of the uterus, inflammations of the pelvic peritoneum and connective tissue, ovarian neurosis, neurotic predisposition, hysteria, and lastly, diseases of the gastro-intestinal canal, especially gastric ulcer, chronic gastritis and constipation.

Morbid changes of the uterus are frequently the cause of reflex neuroses in non-pregnant women. The dependence of gastric disturbances upon the irritability of the uterine nerve-fibers, due to flexion and version of the uterus, to an eroded and congested cervix, to metritis and endometritis, has, in many instances, evidently been proven. Relief has been obtained by the removal of the exciting cause, by the treatment of the uterine diseases after gastric medication had been tried again and again and had failed entirely.

Bearing in mind the physiological changes of the uterus during the pregnant state, its increased functional activity, the influence exerted by the gestation upon the nervous system, and the relation between the neuroses and the disorders of the reproductive organs, so often conclusively proven in non-gravid women, we are compelled to acknowledge the various pathological conditions of the uterus as prominent etiological factors deserving our earnest attention. Cases are on record where the application of caustics to the eroded cervix, scarification of the congested vaginal portion, dilatation of the cervical canal, correction of a flexion, have proven successful in stopping the vomiting, and thus demonstrated the connection between the uterine lesion and the reflex nerve action. In other instances, however, the result of the gynecological treatment has not been so satisfactory, either transitory or no relief has been obtained, and, as a consequence, the influence of the uterine disorders upon the gastric phenomena, their importance as the causative diseases has been questioned.

Undoubtedly it will be often found difficult to decide whether the symptoms result from physiologicol or pathological causes; whether they are due to distention of the uterus or to morbid changes in the sexual organs. All methods of treatment, artificial abortion excepted, may fail to relieve the patient, and she finally may get well by absolute rest and complete abstinence to the surprise of her medical attendant.

Such cases are certainly rare, while, on the other hand, there is abundant clinical evidence of the effect of local treatment. Numerous women have been benefited by the treatment of the uterine lesion; the reflex symptoms have been mitigated or cured by the improvement of the causative disease, and the connection between both has thereby been confirmed.

Attention has been drawn to the importance of endometritis as an etiological factor by F. Veit,* who reported three cases of uncontrollable vomiting, where he was compelled to interrupt pregnancy, and where he found inflammatory processes in the decidua, serotina and vera. Veit believes that by his researches the dependence of hyperemesis upon endometritis is positively proven. As a rule, the endometritis exists prior to gestation, the symptoms are but insignificant, become palpable, however, with the beginning of pregnancy, which frequently is interrupted by this complication. In many instances the endometritis decidua will be found to be the cause of the uncontrollable vomiting; the connection through sympathetic paths must be the same as between gastric disorders and endometritis in non-gravid women. The evidence of an anatomical base, he continues, renders a most careful examination of the uterus imperative, and, if the diagnosis of endometritis, which is very difficult before the removal of the ovum, can be made out, it may be of determining influence as regards the advisability of inducing abortion.

Quite recently E. H. Grandin,† discussing this subject in the New York Obstetrical Society, suggested ovarian neurosis, pressure on unusually hyperesthetic ovaries as a cause of hyperemesis. This view, he says, would be suggested by Dr. Coe's case, which showed that the physiological vomiting of pregnancy could be palliated by teaching the patient to assume the genu-pectoral position before rising, and as often during the day as necessary. He would explain the vomiting of pregnancy, then, by the fact that during the early months the uterus lay low in

---

* *Berliner Clinische Wochenschrift*, 1887, p. 643.

† *American Journal of Obstetrics*, 1890, p. 1382.

the pelvis and pressed on the ovaries; at the third month, when the vomiting usually ceased, the uterus rose above the pelvic brim. In cases of pernicious vomiting it was possible the ovaries were either enlarged through disease or had become impacted between the pelvic brim and the lower uterine segment.

Grandin's theory, though it may be applicable to a given case, will probably not be favorably accepted. To-day the view is predominant that reflex-neuroses may originate in the uterus, and not in the ovary. The removal of normal ovaries for the relief of reflex symptoms is at present restricted to exceptional cases, and it is believed that if a satisfactory result is obtained by the operation, this is due to the changes in the condition of the uterus, to the artificial induction of the menopause, resulting from oöphorectomy. Clinical evidence supports this view.

Grandin's explanation, however, may prove valuable in so far as to induce us to carefully examine the ovaries in cases of hyperemesis. Prolapsed ovaries are by no means a rare affection, but it remains to be demonstrated whether pressure exerted upon them by the enlarged uterus stands in causal relation to gastric disturbance.

Nervous disposition and hysteria, so frequently met with among women of the better classes, add greatly to the discomfort of pregnancy, and, though there are certainly many exceptions, must be considered as prominent predisposing factors of the graver forms of vomiting.

The importance of diseases of the gastro-intestinal canal, especially of gastric ulcers, is emphasized by various authors. According to Horwitz,* "hyperemesis develops in some cases complicated with more or less pathological changes of the stomach and of the intestines. The greater the disturbance in the alimentary canal the easier the ordinary vomiting takes on the character of the uncontrollable form."

The diagnosis of vomiting of pregnancy is by no means as easy as one might think at first sight. While the dependence of this disorder upon the pregnant state may often be determined

* *Praktischer Arzt*, 1882, p. 261.

without much difficulty, cases—especially of the graver forms—may present themselves where this will be found impossible, and where the diagnosis, therefore, must remain doubtful. Faggard* directs our attention to the fact "that so few cases of pernicious vomiting are recorded in German medical literature that the existence of this affection is even questioned." Carl Braun, in a fabulous experience of over one hundred and fifty thousand obstetrical cases, has never observed a single fatal termination. On the other hand, Robert Barnes has himself seen 9 fatal cases. McClintock collected close on 50 cases, and O. W. Doe 48 cases, with 18 deaths, occurring within the last fifteen years, and registered in American and English journals. Gueniot records 118 cases with 46 deaths.

It is not at all improbable, Faggard continues, that the difference of opinion as to the frequency of this disorder between the Germans on the one hand, and the American, French and English observers on the other depends, in a large measure, upon the difference in diagnostic criteria insisted upon by the respective schools. In the majority of the fatal cases of alleged hyperemesis due to pregnancy reported by American, French and English observers, there is a notable absence of reliable records of *post mortem* examinations. In the few cases collected by the Germans, on the other hand, the diagnosis during life has almost invariably been confirmed or negatived by exact investigation of the dead body. Horocks pertinently remarks: "Where there has been no *post mortem* examination in a fatal case of vomiting, I do not think one is entitled to say that pregnancy caused the fatal vomiting. It may have been the cause, and the only cause, or it may have been an aggravation of some other cause, or it may have had nothing to do with it. Scepticism as to the alleged frequency of this disorder in the present state of our knowledge is accordingly eminently in order."

According to Gueniot† three distinct factors are to be taken into consideration in making the diagnosis of vomiting of pregnancy: 1st. The diagnosis of pregnancy. 2d. The diagnosis

* American System of Obstetrics, Vol. I., pp. 411, 415.
† Faggard, American System of Obstetrics, Vol. I., p. 416.

of the adjuvant or determining cause of the vomiting. 3d. The differential diagnosis between obstinate vomiting due to pregnancy and that due to other causes independent of gestation.

It is both interesting and instructive to learn that errors in diagnosis have been made even by eminent clinicians. Thus Faggard* tells us that Trousseau once diagnosticated uncontrollable vomiting, and induced abortion in a case in which the autopsy revealed cancer of the stomach. Beau erred in diagnosis in a case to which the *post mortem* examination showed tubercular meningitis as the probable cause of the vomiting, and Cazeaux narrates the history of a fatal case of alleged hyperemesis of pregnancy where the autopsy disclosed tubercular peritonitis and the absence of pregnancy.

But a mistake in diagnosis is possible even in the other direction, that is, pregnancy may be denied by the patient or not be expected by the physician, and thus be overlooked, as shown in a case recently reported by A. H. Buckmaster.† The patient, a governess in a respectable family, was supposed to be suffering from vomiting due to ulcer of the stomach, and was under treatment two months when she died. In making the autopsy a five months' fœtus was found, but no ulcer whatever, nothing to account for death except the uncontrollable vomiting of pregnancy.

These cases need no comment. I have cited them to demonstrate both the difficulty and the importance of an accurate diagnosis.

It is generally stated that the prognosis of hyperemesis is bad, but this, apparently, is by no means correct. As Faggard justly remarks, "It is doubtful whether an authentic fatal case of this kind is recorded. Such cases have never been seen by observers of the largest experience."

Even the graver forms of this disease yield, as a rule, to rational treatment, unless they are complicated by serious pathological conditions which of themselves render recovery impossible. Pregnancy may aggravate such cases, and perhaps hasten death, but it must be admitted that there exists no causative relation between gestation and the lethal issue.

---

*l. c.

† *American Journal of Obstetrics*, 1890, p. 1381.

A great variety of remedies—still increasing in number every year—have been recommended by different writers. These remedies have proven satisfactory in some cases, but failed entirely in others. This uncertainty of the various methods of treatment, the often but little annoyance caused by the milder forms of vomiting, and the experience that in many instances spontaneous cures occur, have led to the view that interference is not required unless the case presents a more serious aspect. Such advice given in text-books is, at first sight, surprising. Even in mild cases of gastric trouble a careful examination is indicated, and should be insisted upon by the medical attendant, to determine the cause of the disorder, its dependence upon physiological or pathological conditions. Are the generative organs found to be normal? Are there no indications of diseases of other vital organs, especially of the stomach? Is the effect of the vomiting upon the general health but insignificant? It may then be decided whether it be a wise plan to irritate the stomach by various drugs, which, as known from experience, are of so limited value in this reflex affection, or to desist from treatment. It is in this sense, I take it, that such advice has been given, and it is under these circumstances that it deserves recommendation. Nevertheless, such statements in text-books are misleading, fortunately, but to the superficial reader.

While cases of vomiting do well without treatment, diet and regulation of the bowels are usually sufficient to render the gastric disturbances tolerable but the persistent vomiting demands our earnest attention.

Hyperemesis, a reflex neurosis, is due either to physiological changes in the uterus, distention by the growing ovum, or to pathological conditions complicating pregnancy. If we exclude co-existent diseases of the stomach which will be considered later on, it must seem plausible that the treatment should be directed against the causes and not against the symptoms of the gastric disorder; that is, against the uterus, and not against the stomach. The stomach is not the diseased organ. Nausea and vomiting of pregnancy are only the symptoms of some functional disturbance of the nervous system, originating in the uterus, like the nausea and vomiting of sea-sickness, an analogous disease, dependent upon the motion of the ship. For this reason gastric medications must fail to favorably influence hyperemesis; for this reason none of the innumerable remedies recommended are found to be reliable—some of them are worse than useless.

There are three classes of cases, however, which sometimes may be relieved by the administration of drugs, viz.: 1st, Women, who prior to gestation, have been afflicted with diseases of the stomach, as chronic gastritis and gastric ulcer; 2d, women of an unusual nervous irritability; and 3d, hysterical women.

In cases of the first category sub-nitrate of bismuth, bicarbonate of sodium, Carlsbad water, oxalate of cerium, the tincture of nux vomica, etc., may be tried and may sometimes be found of decided value, while the nervines and sedatives may give relief to nervous and hysterical women. Opium and its preparations, the bromides and chloral, either administered by the mouth, by the rectum, or hypodermically, as the circumstances require, are the medicinal agents which have the best reputation, and which, in these cases, sometimes successfully depress the reflex irritability, and thus alleviate the symptoms. Blisters, the application of chloroform, ether, and of the faradic current to the epigastrium, of the ice-bag to the dorso-lumbar region have been tried and have afforded relief in some instances.

The resort to local treatment is indicated in all those cases in which a morbid condition of the uterus has been made out. Retroflexion and retroversion are to be corrected, and, if necessary, the uterus is to be retained in position by a suitable pessary. A congested vaginal portion may be relieved by scarifications, while the application of carbolic acid, or of a 10 per cent. solution of nitrate of silver to the eroded cervix will often prove successful in mitigating the distressing symptoms. Faggard* states that in Vienna a 10 per cent. solution of nitrate of silver is employed in all cases of severe vomiting, irrespective of the condition of the vaginal portion. "The weight of testimony in favor of this simple procedure, collected from innumerable sources, is so great as to make its employment absolutely obligatory before resorting to more radical methods."

Dilatation of the cervix—Copeman's method—has proven successful according to some writers, while others report negative results. In the only case of severe vomiting which I have observed it had a most remarkable effect. The nausea disappeared instantly, but only for a few hours. The method was again applied, but no result was obtained the second time.

Horwitz† recommends that in the severer cases of vomiting the patient should be placed at rest in bed in the horizontal position, that the room be darkened, and that, if the stomach rejects everything, rectal alimentation should be resorted to. Crushed ice to quench the thirst is allowable. I can fully endorse this plan.

When these various methods have failed, when the vomiting actually is uncontrollable and seriously endangers the patient's life, the induction of abortion or premature labor is indicated, and will, if done in time, to a certainty save the woman.

